# Fabricating a robust POSS-PCL nanofiber scaffold for nesting of mesenchymal stem cells: potential application in bone tissue regeneration

**DOI:** 10.1186/s13036-022-00317-5

**Published:** 2022-12-21

**Authors:** Leyla bagheri, Hasan Valizadeh, Kazem Dindar-safa, Nosratollah Zarghami

**Affiliations:** 1 Department of Chemistry, Faculty of Sciences, Azarbaijan Shahid Madani University, 53714-161 Tabriz, Iran; 2 Department of Clinical Biochemistry and Laboratory Medicine, Faculty of Medicine, Tabriz University of Medical Sciences, Tabriz, Iran; 3 Organosilicon Research Laboratory, Faculty of Chemistry, University of Tabriz, Tabriz, 5166616471 Iran; 4 Department of Medical Biochemistry, Faculty of Medicine, Istanbul Aydin University, Istanbul, Turkey

**Keywords:** Electrospinning, Mesenchymal stem cell, Nanocomposite, Polyhedral oligomeric silsesquioxane, POSS-PCL, Tissue engineering

## Abstract

**Background:**

According to recent studies, electrospun Poly (Ɛ-caprolactone) (PCL) is an absorbing candidate for the formulation of biocompatible scaffolds used in tissue engineering. Tissue engineering is a set of techniques for producing or reconstructing tissue, whose primary purpose is to restore or improve the function of tissues in the human body. Tissue engineering combines the principles of materials and cell transplantation to develop alternative tissues or promote endogenous regeneration. However, this electrospun scaffold, consisting of PCL, has disadvantages such as low cell adhesion, inactivity of the surface, osteoinduction, and acidic destruction of the scaffold that causes inflammation at the implant site, often making it unsuitable implant. This study aimed to improve PCL base cellular scaffolds with the formulation of polyhedral oligomeric silsesquioxane – Polycaprolactone (POSS-PCL) nanofiber scaffolds. The present research focuses on the synthesis of nanofibers for their cell interaction features, and application in bone tissue engineering and regeneration.

**Results:**

POSS/ PCL Nanocomposites with 2, 5, and 10 wt.% of POSS were synthesized in the Trichloromethane, then POSS – PCL Nanofibers were prepared by the electrospinning technique. In this study, the structures of nanohybrids and nanofibers have been evaluated by FTIR, HNMR, XRD, SEM, EDX, and DSC. The biocompatibility of formulated POSS-PCL scaffolds was detected using mesenchymal stem cells (MSCs). Then several parameters were examined, involving DCFH ROS detection system, gene expression (cell viability/apoptosis, osteogenesis potentiality, and redox molecular homeostasis.

**Conclusions:**

Based on our results, POSS-PCL nano-scaffolds in comparison with PCL have shown a robust potentiality in homing, growth, and differentiation of stem cells.

**Graphical Abstract:**

Synthesis of POSS-PCL Nanofibers and their potential application in Bone Regeneration.

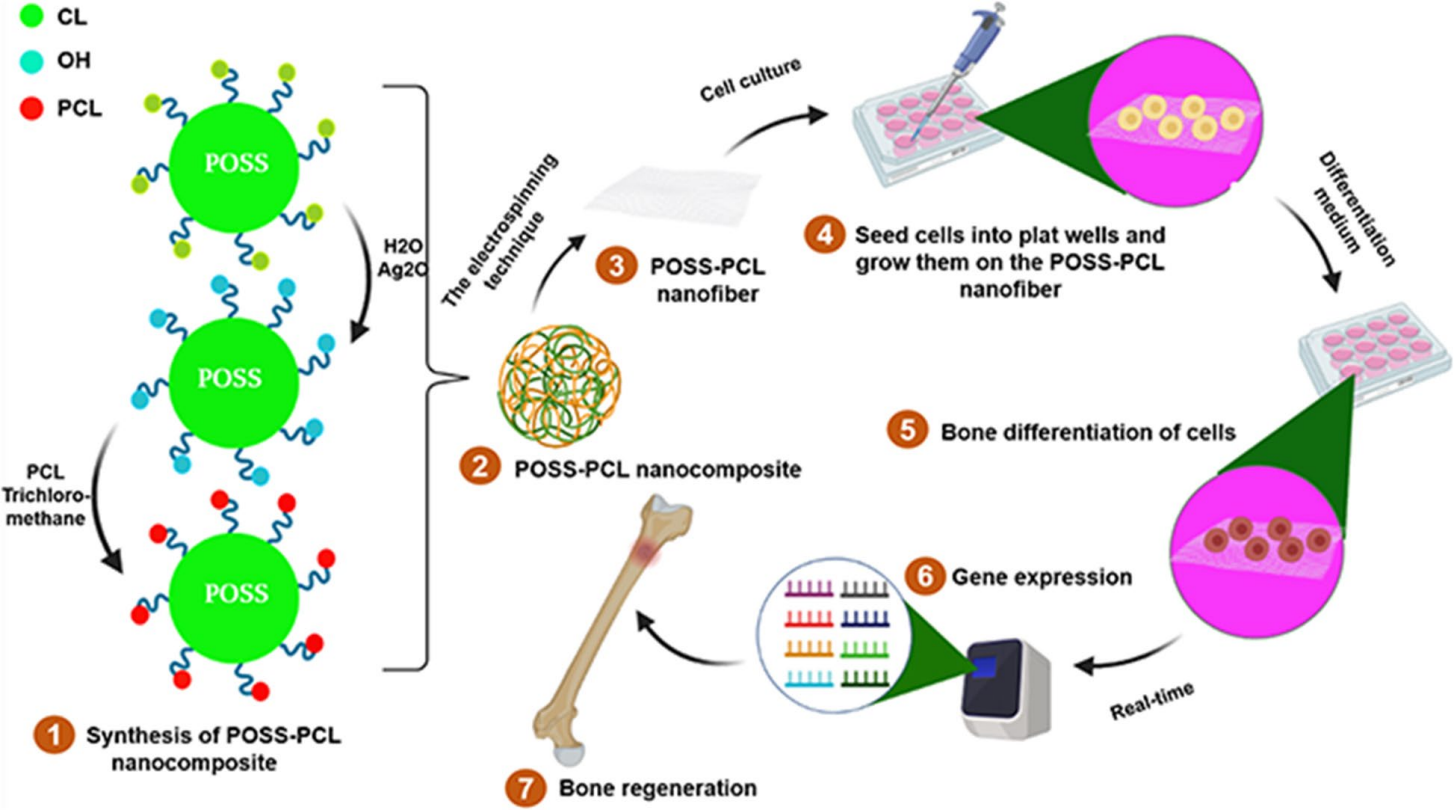

## Introduction

Most bone diseases, such as bone infections and bone tumors, require bone regeneration. However, bone injuries as a major problem still overshadow the normal life of patients and require replacement bone grafts for regeneration. Accordingly, tissue engineering, known as the design and fabrication of new tissue to restore the function of defective organs or lost tissue, emerged in the early 1990s to compensate for these limitations. Tissue engineering (TE) is the multidisciplinary field of designing a therapeutic product that utilizes the combination of matrix scaffolds with viable human cell systems, for the repair or regeneration of damaged tissues [1–4]. Tissue engineering requires appropriate stem cells, an optimal culture system, and nano-surface nano scaffolds with proliferation or differentiation chemo-physical [5–7]. The surface topology, mechanical factors, and chemical components of nano-scaffolds affect the quality of the engineered tissue [8–10]. So, the development of scaffolds to provide optimal cultural conditions remains an active field of research. On the other hand, bone tissue is a cell with a composite structure that includes organic and inorganic parts. For this purpose, we propose POSS polymers, in which case Polyhedral Oligomeric Silsesquioxane (POSS) is a new generation of nanocomposite materials with high biocompatibility and superior mechanical properties, which has been successfully used as a scaffold for the production of synthetic organs as well as a coating for medical instruments. POSS molecules are chemical compounds with a particular structure. Their structure is either cube-shaped or cage-like [11]. The cage-like structure is formed by Si and O atoms, and due to their solid bond of Si-O (809 KJ/mol), they have high strength [12, 13]. Besides Si atoms that are at the vertices of the cube and the O atoms on the sides of the cube between the two vertices, the organic functional groups are located on the Si -vertices through the covalently bond. These functional groups surround the cubic structure and thus, form a POSS cage structure [14]. These molecules are called organic-inorganic hybrids since they are made up of inorganic cores (Si) and organic groups [15]. Considering the properties of these cubic compounds in recent years, great attention has been paid to these POSS compounds in technology and science [16, 17]. The main properties of these POSS compounds are biocompatibility, non-toxicity, high thermal and mechanical stability, as well as their surface properties, which allow them to be used in making polymers and copolymers by forming a bond between them and polymers such as Poly (Ɛ-caprolactone) (PCL), Polyethylene Glycol (PEG), Polylactic Acid (PLA), etc. Recently, scaffolds with nanofibrous architecture, fabricated by electrospinning technique, have been significantly utilized as a template for cell proliferation and developing functional tissue engineering. The mechanical properties of POSS-PCL nanocomposite films from other POSS derivatives have been investigated and described in an article by Mónica Cobos and her colleagues [18]. Diameters in the nanometer range, high porosity, and the high surface-area-to-volume ratio of nanofiber highly match the morphology and function of extracellular matrices, providing a proper biomimetic scaffold for cell recognition, attachment, proliferation, and differentiation as well as drug loading [19–21]. They form composites that, when their performance is improved, can be used for biomedical purposes in biotechnological fields such as biomedical devices, drug delivery systems, tissue engineering products, dental composites, biosensors, etc. [22–24]. Cubic POSSs are ideal starting materials for nano-production; therefore, they can be used as a new class of nano-fillers to produce high-performance nanostructured composites. According to research, POSS-polymer nanocomposites possess advanced mechanical features, thermal constancy, oxidative and anti-ignition endurance, and surface hardening [25, 26]. It must be noted that the improved properties of polymer-POSS nanocomposites depend on several factors, such as the chain measurement of the functional groups joined to the silicon atoms, and the polymer chains that bind the covalent bonds with the nanoparticles, and the preparation techniques [27–30]. PCL is among the common polymers that are used to make POSS-containing nanocomposites [31, 32]. PCL is a key aliphatic polyester due to its degradability, biocompatibility, and non-toxic properties that are focused on by researchers and are utilized by the FDA in biomedical applications [33]. However, the effects of these substances are still unknown. Goffin et al. showed that well-dispersed POSS nanoparticles in the nanocomposites are achievable only when that POSS-g-PCL nanohybrid is used as a masterbatch [31, 34]. Miltner et al. fabricated the POSS nanocomposites based on PCL in the presence of aminopropylheptakis (isobutyl)- POSS and found that dispersion quality is achieved when the nanocomposite is prepared by in situ polymerization [35]. Moreover, they showed that a greater grafted chain length was more effective in refining the compatibility between POSS and the PCL matrix. The morphology and crystallization of PCL/octaisobutyl-POSS nanocomposites, made by the solution casting technique, were examined by Pan et al., and aggregates of submicron-sized POSS particles were obtained and enhanced the crystallization of PCL [36]. In recent studies, the morphology and crystallization of PCL/octaisobutyl-POSS nanocomposites fabricated by solution casting procedure were investigated and the enhancement of crystallization of PCL and aggregates of submicron-sized POSS particles were depicted [37]. Prior studies have shown that POSS-incorporated hybrid polymers possess good cytocompatibility and support the attachment, spreading and proliferation of chondrocytes. Hence, POSS is the potential candidate to fabricate hybrid polymers with enhanced and controlled properties. Interestingly, POSS- enhanced natural polymers are only an emerging field, and their application in bone repair and regeneration has to the best of our knowledge not been widely proposed, especially in repairing connective tissue bone defects in vivo [38]. Recently, natural polymer-based nanocomposites have come into prominence for bone tissue scaffold design. As a natural polymer, PCL have promising characteristics such as non-toxicity, non-allergenicity, mucoadhesivity, biocompatibility and biodegradability. The ease of processing of PCL into porous structure is another promising characteristic to fabricate a variety of scaffolds. However, it has a limitation with mechanical properties compared to natural bone. Thus, PCL is reinforced with an inorganic filler in order to overcome this limitation. Nanofillers provide dramatic improvements in physical properties (thermal stability, mechanical properties, swelling behavior) of polymer matrix and surface morphology by altering the structure at micro- nanoscale [39]. Besides, recently bioactive nanofillers have come into prominence in scaffold designs for bone regeneration to provide mimicry with bone structure. POSS, regarded as the smallest silica particle, is widely used as nanofiller in polymer systems. POSS-based nanocomposites are deduced as novel materials having potency for biomedical applications owing to the enhanced biocompatibility and physicochemical characteristics [40]. The aim of this work was to integrate the beneficial features of PCL and POSS nanoparticle to design nanofiber for bone tissue regeneration.

Biologically, it escapes the osteogenic power of several human AD-MSCs. AD-MSCs are a cell crowd with multi-differentiation potential that were initially identified in the BM-MSCs more than 40 years ago [41, 42]. AD-MSCs not only have therapeutic properties but also the ability to differentiate significantly. They can be separated from all major layer derivatives, including cartilage, bone, nerve cells, adipose tissue, and cardiomyocyte [43]. These AD-MSC-based healing properties are an excellent treatment tactic for wound curing and tissue regeneration [44]. This study tried to improve PCL based scaffolds with the formulation of POSS-PCL nanofiber scaffolds for tissue engineering applications. The case that has increased the investigation in this field is the possibility of modifying PCL surfaces by means of POSS molecules by the electrospinning method. Electrospinning is one of the most important methods of preparing polymer nanofibers. The electrospun felt product is a type of nanofiber that creates a thin layer on the collecting metal plate during the electrospinning process. In fact, this layer results from the freezing or incomplete freezing of the jet on two-dimensional planes. Because this layer has a mesh structure under the electron microscope, it is called nanofiber mesh or nanofiber network. To describe this scaffold and its effect on cells, we employed DCFH ROS discovery system, gene expression (RUNX-2, Osteocalcin, Nrf2, BAX, VEGF gens), and apoptotic techniques. Based on the outcomes, the biocompatibility and bone differentiation ability of the scaffold affirmed its applicability in bone tissue engineering.

## Methods and materials

### Materials

Methanol, Chloride acid, ethanol, Tetrahydrofuran (THF), Trichloromethane (DCM), Dichloromethane, and Dimethyl Sulfoxide (DMSO) were purchased from Sigma-Aldrich (St. Louis, MO, USA). (3-chloropropyl) trimethoxysilane, di-n-butyltin dilaurate, and PCL (Mn= 80000), DMEM, Beta glycerol phosphate, Dexamethasone, ascorbic acid, Penicillin Streptomycin antibiotic, Rhodamine B (RhoB), H_2_O_2_, and Trizol were bought from Sigma-Aldrich (Steinem, Germany). Fetal Bovine Serum (FBS) and Trypsin-EDTA were also purchased from Gibco (USA). Thermo Scientific RevertAid First Strand cDNA Synthesis Kit was purchased from Thermo Fisher Scientific Inc. Mesenchymal stem cell (Pasteur Institute, Iran).

### Synthesis of octa(3-chloropropyl) POSS [(ClC_3_H_6_)_8_Si_8_O_12_]

First, we prepare a round bottom flask and two openings containing a magnetic stirrer. Then we attach an increasing funnel to one of the openings of the flask. We mount a condenser on the flask. Now, pour 700 cc of dry methanol and 23.3 cc of concentrated chloride acid into a flask and turn on the stirrer to stir the solution. Add 70 gr (0.35 mol) of (3-chloropropyl) trimethoxysilane drop by drop while stirring vigorously. Allow the stirring to continue for 3 hours. Then turn off the mixer to keep the mixture in the flask at room temperature. After 43 hours, add 0.69 gr (1.104 mmol) of di-n-butyltin dilaurate catalyst with vigorous stirring and allow to stir for 2 hours. Then we turn off the stirrer and after 48 hours, colorless crystals are obtained. We collect the crystals and do the filtration operation and wash them three times with methanol. We put it in the oven for 48 hours at 60 °C to dry, and the product yield is 10.6 grams.

### Synthesis of octa (3-hydroxypropyl) POSS [(HOC_3_H_6_)_8_Si_8_O_12_]

Pour 6 gr of octa (3-chloropropyl) POSS into a round bottom flask containing a magnetic stirrer, then add 150 cc of ethanol and 150 cc of tetrahydrofuran and turn on the stirrer to stir the contents of the flask to form a transparent solution. Then add 6 gr of freshly prepared silver oxide with 1cc of deionized water to the contents of the flask, then reflux is done in the dark with vigorous stirring for two days. After two days, we filter the impurities in the contents of the flask and perform the filtration operation three times, then we put the solution in a rotary to obtain white deposits by evaporating the solvent. The resulting sediments are dried in an oven at 60 ° C for 48 hours.

### Synthesis of POSS/ PCL Nanocomposites

POSS compounds, with 2, 5, and 10 wt.%, were prepared in the Trichloromethane. POSS and PCL (15 wt.%) solutions were mixed in pairs with the water bath system for 1 hour. The resulting solutions were poured into glass plates to evaporate the solvent and to obtain a film. The resulting films were dried in a vacuum oven at 60 degrees for 3 days. The produced film was a POSS-PCL nanocomposite.

### Synthesis of POSS – PCL Nanofibers

The electrospinning technique was applied for the synthesis of POSS- PCL nanofibers. Therefore, a 3 ml syringe containing POSS- PCL (15 wt.%) solution was placed on the syringe pump, and a 15cm×10cm aluminum foil was located in the system as a collector, and the necessary parameters including distance, voltage, and injection were adjusted, and then the electrospinning device turned on. To achieve a layer-by-layer POSS- PCL membrane, the parameters were repeated at all stages, constantly. In this process, distance=14cm, voltage=15kV, injection=2 ml/h were selected [45].

### Chemical characterization

#### Fourier transforms infrared (FTIR) spectroscopy

Chemical analysis of nanofibers and nanocomposites was performed by an FTIR device (Bruker, VECTOR 22, Germany). In all cases, 21 scans were utilized to record the spectra. The spectrum of the hybrid nanocomposites and nanofibers was recorded from 400 to 4000 cm^-1^.

#### Proton Nuclear Magnetic Resonance (^1^H NMR) spectra

^1^H NMR spectra of the synthesized hybrid compounds and nanofibers were gauged on a Bruker 250 MHz Ascend spectrometer (Bruker Biospin GmbH, Germany). Samples were soluble in deuterated chloroform (CDCl_3_).

#### X-ray diffraction (XRD)

The XRD curves of synthesized hybrid compounds and nanofibers were registered by a Shimadzu X-ray diffractometer (model Lab XRD-600) with Cu K_α_ irradiance (λ= 1.5418 Å) at 40 kV and 30 mA in the 2θ range of 0° < 2θ < 60° with a scan speed of 10º/min.

#### Scanning electron microscopy (SEM) analysis

The morphology and diameters of electrospun hybrid compounds and nanofibers were investigated by SEM device (MIRA3, TESCAN, Czech) after sputter covering with gold. The Average Fiber Diameter (AFD) data were collected from 30 fibers by an image analysis software (Image J 1.42q, National Institute of Health, USA).

#### Energy dispersive X-ray (EDX) analysis

The atoms in the hybrid compounds and nanofibers were identified by the EDX device (VEGA TESCAN, XMU, USA).

#### Differential scanning calorimetry analysis

DSC analyses were performed by a Thermo Analysis (TA) device TA- DSC 822 e (Mettler Toledo, Swiss) with a heating rate of 10 ºC min^-1^ and under nitrogen gas. The nanofibers (about 4 mg) were warmed from -30 to 300 ºC and kept in the molten situation for 5 min (to remove the thermal history) then, they were chilled to -60 ºC at 10 ºC min^-1^ and kept in a cold place for 5 min, and reheated to 100 ºC at 10 ºC min^-1^.

### Cell loading and bone differentiation

Mesenchymal stem cells (MSCs) were seeded in DMEM media, with 10% FBS. Then 70 to 80% of confluence, the cells were collected by trypsinization. By placing the POSS-PCL scaffolds at the bottom of each well of the 6-well plate, the cells are seeded on the designed scaffolds for the differentiation. To investigate changes in gene expression pattern, the culture was performed in a differentiation medium for 3 weeks and cells were examined on differentiation days 7, 14, and 21. Also, on the 21st day of differentiation, Alizarin red S staining was used to evaluate the bone differentiation of mesenchymal stem cells. The medium used to culture the cells at this stage is the bone differentiation medium, which contains 10% FBS, 500 μl of streptomycin-penicillin, 0.2 mM ascorbate, and 0.1 μM dexamethasone and 10 mM b-glycerol phosphate. The bone differentiation medium was refreshed every 2 days with the freshly prepared medium. In this study, we had a control plate in all stages, which was without a scaffold, and in other plates, the bottom of the plates was completely covered with nanofibers, and the walls were slightly covered from the sides. In this way, we tried to prevent cells from entering under the nanofibers as much as possible. In Figs. 6 and 7, the control plates have also been analyzed with the plates containing the scaffold.

### Biocompatibility test for designed scaffolds

#### DCFH-DA assay for measurement of intracellular free radicals

After 24 h, the cells cultured on scaffolds were pre-treated with 10 µM of dichloro-dihydro-fluorescein diacetate (DCFH-DA) and incubated at 37°C for 1 h. After discarding the medium, the MSCs were washed (3x) with PBS solution and trypsinized. The cells were monitored using Cytation™ 5 cell imaging instrument (BioTek, Winooski, USA) .

#### Analyzing of apoptotic population

Apoptosis FITC Annexin- V kit was used to evaluate the planned process of cell death by flow cytometry. Differentiation between apoptotic and necrotic cells can be accomplished by co-staining with propidium iodide (PI). Therefore, FITC Annexin- V is used as a marker for phosphatidylserine and PI as a marker for dead cells. The cells were implanted in a plate of 6 wells with a density of 3×10^5^ cells. After 21 days, a series of cell lines were treated with H_2_O_2_ and then incubated for 24 hours. After trypsinization and collection and centrifugation of cells, cells were washed with PBS. Now 100 microliters of dilute binding buffer were added to the cells, followed by are added 5 microliters of Annexin- V and 5 microliters of PI to the cell suspension and incubated for 20 minutes at room temperature in the dark. Finally, phosphatidylserine binding to Annexin- V was used as an indicator for flow cytometry analysis to quantify the percentage of apoptotic cells.

### Q-PCR and characterization of RNA profile

Total RNAs were extracted from the MSCs after 7, 14, and 21 days via Trizol. The RNA purity and yield were revealed via NanoDrop, ND-1000 spectrophotometer (NanoDrop Technologies, Inc., Wilmington, USA). Then, the cDNA was produced matching to the literature. Real-time PCR reactions were done for bone differentiation genes, the major antioxidants transcription factors Nrf2 and the gens involved in cell proliferation and apoptosis. In qPCR, the level of statement was determined based on the PCR cycle amount (Ct). The endogenous regulator GAPDH was employed for the standardization of mRNA levels. The Ct quantities were applied to compute relative expression thru SPSS software (ver 14.0) by the difference in the Ct quantities of the goal RNAs after the standardization to the RNA input surfaces. Comparative quantification was signified matching to the Pfaffl method [46]. Each reaction was done in triplicate. The primer sequences are shown in Table 1.

**Table 1:** Sequence of the oligonucleotides for real-time PCR

**Gene**	**Sequence (5´→ 3´)**
**BAX**	F 5´ GATGCGTCCACCAAGAAG 3´
	R 5´ AGTTGAAGTTGCCGTCAG 3´
**Nrf-2**	F5´- AGACAGGTGAATTTCTCCCAAT-3´
	R5´- TTTGGGAATGTGGGCAAC- 3´
**VEGF**	F5’- CACCACCGACAGAACAGTCC -3’
	R5’-CGAATCCAATTCCAAGAGG-3’
**Osteocalcin**	F 5´ ACAAGAGATTCAGCGACT-3´
	R5´-GGTTCTTGGCTTCCTGTTTC-3´
**RUNX-2**	F5´- CAGACCAGCAGCAGCACTCCATA-3´
	R5´- CAGCGTCAACACCATCATTC-3´
**GAPDH**	5′-AAGCTCATTTCCTGGTATGACAACG
	5´ TCTTCCTCTTGTGCTCTTGCTGG 3´

### Statistical investigation

Data were stated as average quantities with standard deviance (S.D.) from three autonomous experiments. Statistical significances among groups were examined by one-track ANOVA followed by Dunnett’s manifold comparisons *post hoc* test. The significant amount was a *p*-value of <0.05.

## Results

### Structural characterization of POSS-Cl

**Fig. 1:**
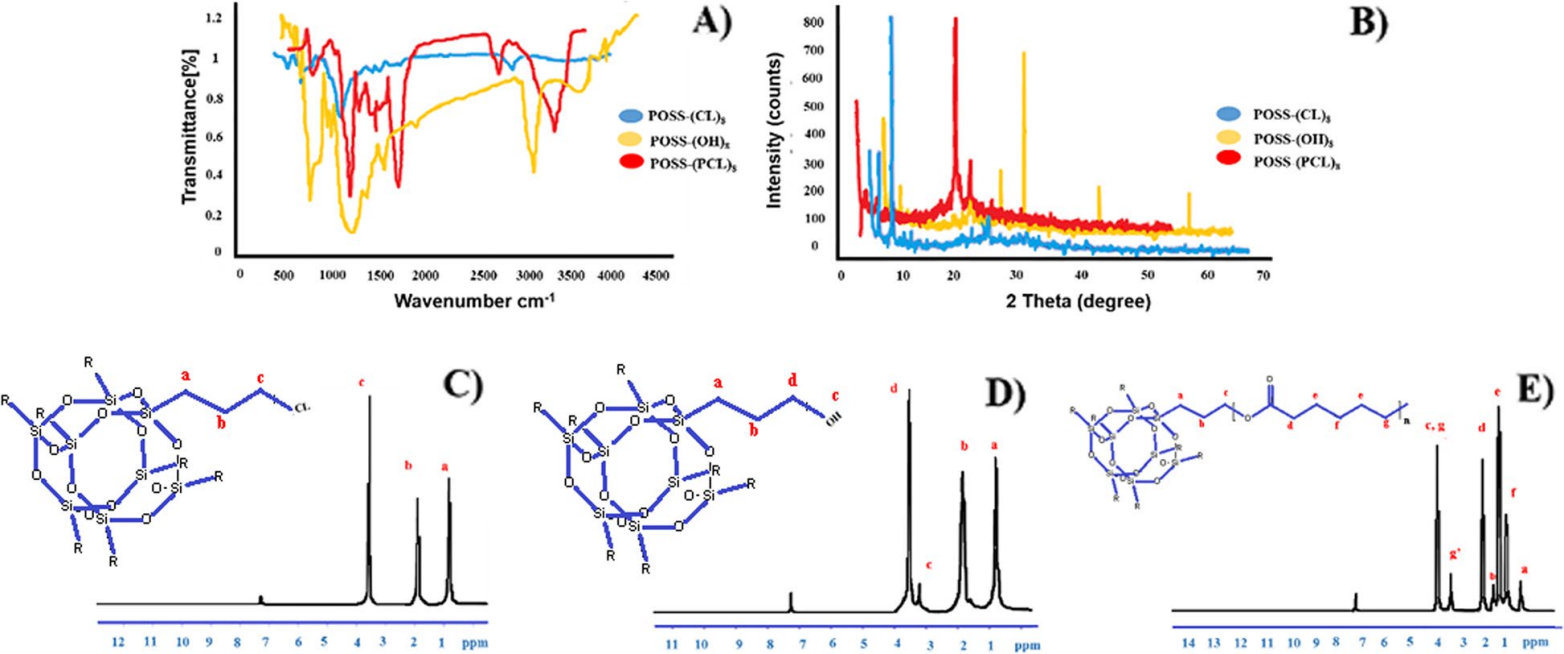
**A** FTIR spectra of POSS-(Cl)_8_, POSS-(OH)_8_ nanoparticles, and POSS-(PCL)_8_ nanofibers, **B** XRD spectra of POSS-(Cl)_8_, POSS-(OH)_8_ nanoparticles, and POSS-(PCL)_8_ nanofibers, **C**^1^ H NMR spectra of POSS-(Cl)_8_, **D**^1^ H NMR spectra of POSS-(OH)_8_, **E**^1^ H NMR spectra of POSS-(PCL)_8_

**Fig. 2:**
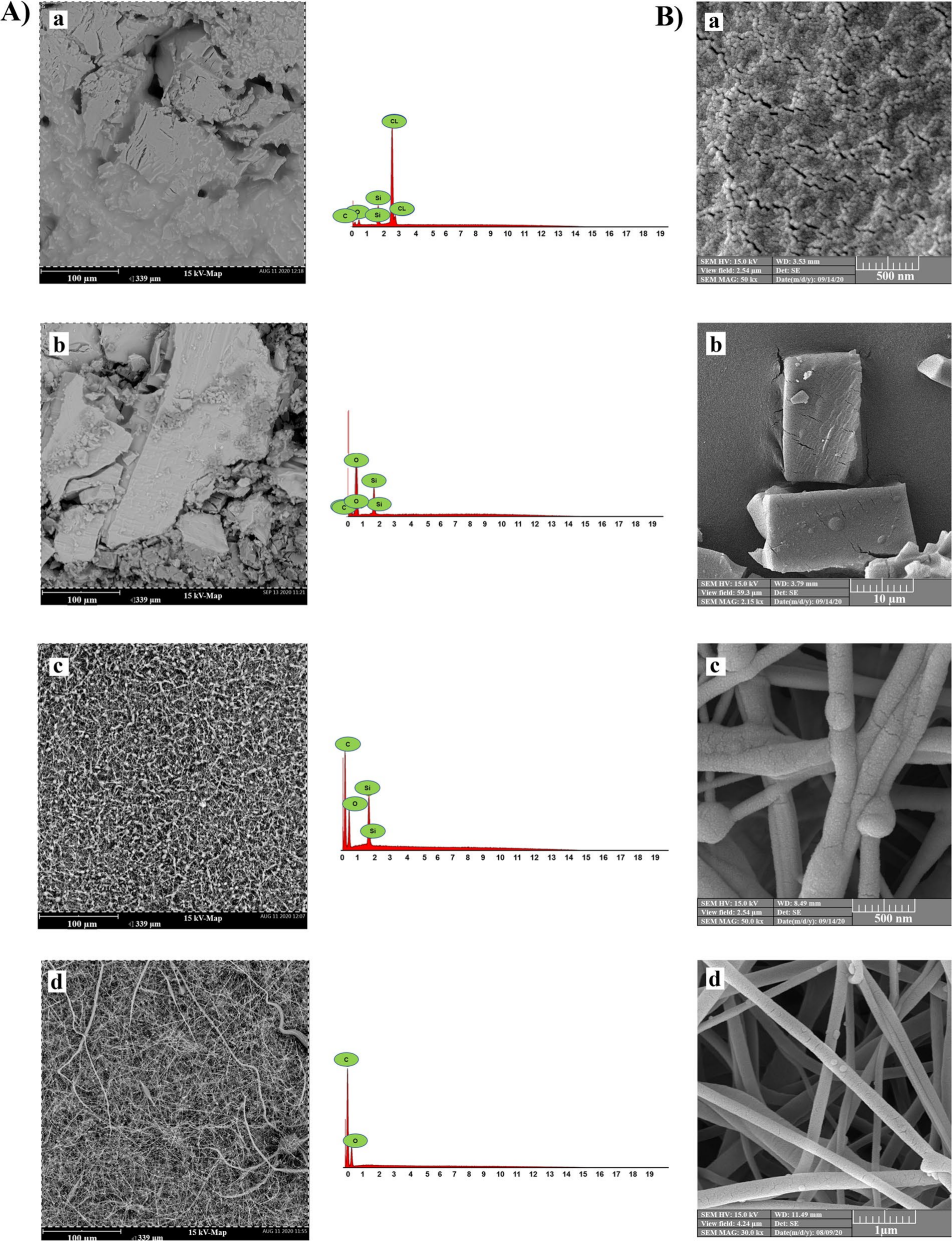
**A** EDX image and spectrum of, a) POSS-(Cl)_8_, b) POSS-(OH)_8_, and c) POSS-(PCL)_8_, and d) PCL. The EDX spectrums show the constituent elements of each compound. **B** SEM images of a) POSS-(Cl)_8_, b) POSS-(OH)_8_, c) POSS (5%) -(PCL)_8,_ and d) PCL. These images clearly show the cubic structure of POSS-(Cl)_8_ and POSS-(OH)_8_ nanoparticles and of PCL and POSS-(PCL)_8_ nanofibers, comparison of c and d nanofibers shows the binding of POSS nanoparticles to PCL.

The FTIR spectroscopy of POSS-(Cl)_8_ is displayed in Fig. 1A. The identified peaks in FTIR spectrum (KBr window, cm^-1^) are: ν(CH) 2984, 2954, 2872; ν(SiOSi) 1109; ν(CCl) 698. X-ray diffraction curves show POSS-(Cl)_8_ crystallinity (Fig. 1B). Three distinct peaks are observed in 2θ = 7.3º, 9.2º, and 21.8ºfor POSS-(Cl)_8_. Also, the structure of POSS-(Cl)_8_ was confirmed by ^1^HNMR spectroscopy (CDCl_3_, 298 K, 250 MHz; ppm) (Fig. 1C). The chemical interpretation of protons in this compound is as following: 0.76-0.83 (t, SiCH_2_, 16H, ^3^J_HH_ = 7.5 Hz); 1.8-1.9 (m, CH_2_, 16H, ^3^J_HH_ = 7.5 Hz); 3.5-3.56 (t, CH_2_Cl, 16H, ^3^J_HH_ = 7.5 Hz). EDX tests confirmed the presence of C, O, Si, and Cl elements in the POSS-(Cl)_8_ and determines their ratio (Fig. 2A.a). SEM tests are utilized to monitor the structural evolution of these materials. The SEM image shows a large number of nanoparticles of POSS-(Cl)_8_ with an average diameter of about 40±5 nm (Fig. 2B.a), which composes the cubic structure of POSS-(Cl)_8_.

### Structural characterization of POSS-OH

The FTIR spectroscopy of POSS-(OH)_8_ is displayed in Fig. 1A, the identified peaks in the FTIR spectrum (KBr window, cm^-1^) are ν(OH) 3402; ν(CH) 2875-2956; ν(SiOSi) 1121; ν(C-O) 700. X-ray diffraction curves show POSS-(OH)_8_ crystallinity (Fig. 1B). Three distinct peaks were observed in 2θ = 21.8º, 27.6º, 31.9, and 45.9º for POSS-(OH)_8_. Also, POSS-(OH)_8_ structure was confirmed by ^1^HNMR spectroscopy (CDCl_3_, 298 K, 250 MHz; ppm) (Fig. 1D). The chemical interpretation of protons in this compound is as following: 0.76-0.83 (t, SiCH_2_, 16H, ^3^J_HH_ = 7.5 Hz); 1.8-1.9 (m, CH_2_, 16H, ^3^J_HH_ = 7.5 Hz); 3.39 (s, OH, 8H) 3.5-3.56 (t, CH_2_OH, 16H, ^3^J_HH_ = 7.5 Hz). EDX tests confirmed the presence of C, O, and Si elements in the POSS-(OH)_8_ and determined their ratio (Fig. 2A.b). SEM tests were used to monitor the structural evolution of these materials. The SEM image showed a large number of nanoparticles of POSS-(OH)8 with an average diameter of about 40±4 nm (Fig. 2B.b), which composes the cubic structure of POSS-(OH)_8_.

### Structural characterization of POSS-PCL

**Fig. 3:**
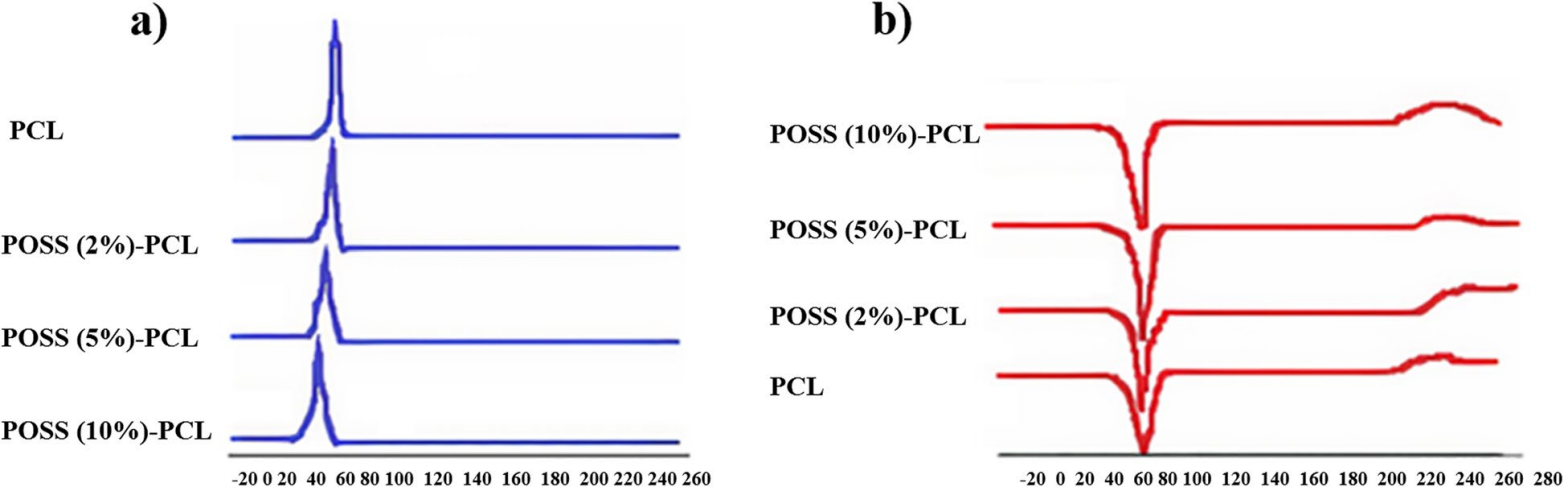
Differential Scanning Calorimetry analysis (DSC) PCL, POSS (2%)-PCL, POSS (5%)-PCL and POSS (10%)-PCL nanofibers. **a** crystallization temperature, **b** melting temperature

The FTIR spectroscopy of POSS-(PCL)_8_ is displayed in Fig. 1A, the identified peaks in FTIR spectrum (KBr window, cm^-1^) are: ν(OH) 3614; ν(CH) 2893-2941; ν(C=O) 1730; ν(SiOSi) 1110; ν(C-O) 688. X-ray diffraction curves showed POSS-(PCL)_8_ crystallinity (Fig. 1B). Three distinct peaks were observed in 2θ = 21.5º, 22º, and 24º for POSS-(PCL)_8_. Also, POSS-(PCL)_8_ structure was confirmed by ^1^HNMR spectra. (CDCl_3_, 298 K, 250 MHz; ppm) (Fig. 1E) spectroscopy. The chemical interpretation of protons in this compound is as follows:

0.76-0.83 (SiCH_2_-, ^3^J_HH_ = 7.5 Hz), 1.37-1.42 (OOCCH_2_CH_2_CH_2_-, ^3^J_HH_ = 7.5 Hz), 1.61-1.66 (OOCCH_2_CH_2_CH_2_CH_2_-, ^3^J_HH_ = 7.5 Hz), 1.86 (SiCH_2_CH_2_-, ^3^J_HH_ = 7.5 Hz), 2.27-2.32 (OOCCH_2_-, ^3^J_HH_ = 7.5 Hz), 3.50-3.56 (OOCCH_2_CH_2_CH_2_CH_2_CH_2_-, ^3^J_HH_ = 7.5 Hz), 4.02-4.07 (-CH_2_OOCCH_2_CH_2_CH_2_CH_2_CH_2_-, ^3^J_HH_ = 7.5 Hz). EDX tests confirmed the presence of C, O, and Si elements in the POSS-(PCL)_8_ and evenly scattering POSS and its presence in POSS-PCL (Fig. 2A.c). SEM tests were used to monitor the structural evolution of these materials. The SEM image showed a large number of nanofibers of POSS (5%) -(PCL)8 with an average diameter of about 100±5 nm (Fig. 2B.c), and binding of POSS nanoparticles to PCL fibers. The thermal behaviors of PCL and POSS-PCL were investigated by DSC analysis. In DSC thermograms, POSS-PCL nanofibers are not degraded in the temperature range of -30 to 230 ° C, but they had thermal degradation in the range of approximately 238-242 °C. The melting temperature, crystallization temperature, and melting and crystallization enthalpy of PCL and POSS-PCL nanofibers were reported in Table 2. Through DSC thermograms, it can be concluded from these results that the binding of the POSS molecule does not reduce the quality of the nanofibers, but to some extent, it increases the thermal stability of the nanofibers. From the data examined in Table 2 it can be concluded that by an increase in melting temperature and crystallization temperature and enthalpy in nanofibers containing POSS, the crystallinity of these nanofibers become higher when compared to PCL nanofibers (Fig. 3).

**Table 2:** Thermal features of nanofiber

**Nanofiber**	**T**_**c**_** (ºC)**	**T**_**m**_** (ºC)**	**ΔH**_**m**_** [J/g]**	**ΔH**_**c**_** [J/g]**
**PCL**	26.94	62.13	-85.50	73.87
**POSS (2%)-PCL**	24.51	63.12	-69.87	43.71
**POSS (5%)-PCL**	20.44	63.82	-66.86	40.65
**POSS (10%)-PCL**	19.63	62.68	-56.37	33.05

### Osteogenesis and Alizarin red staining

**Fig. 4:**
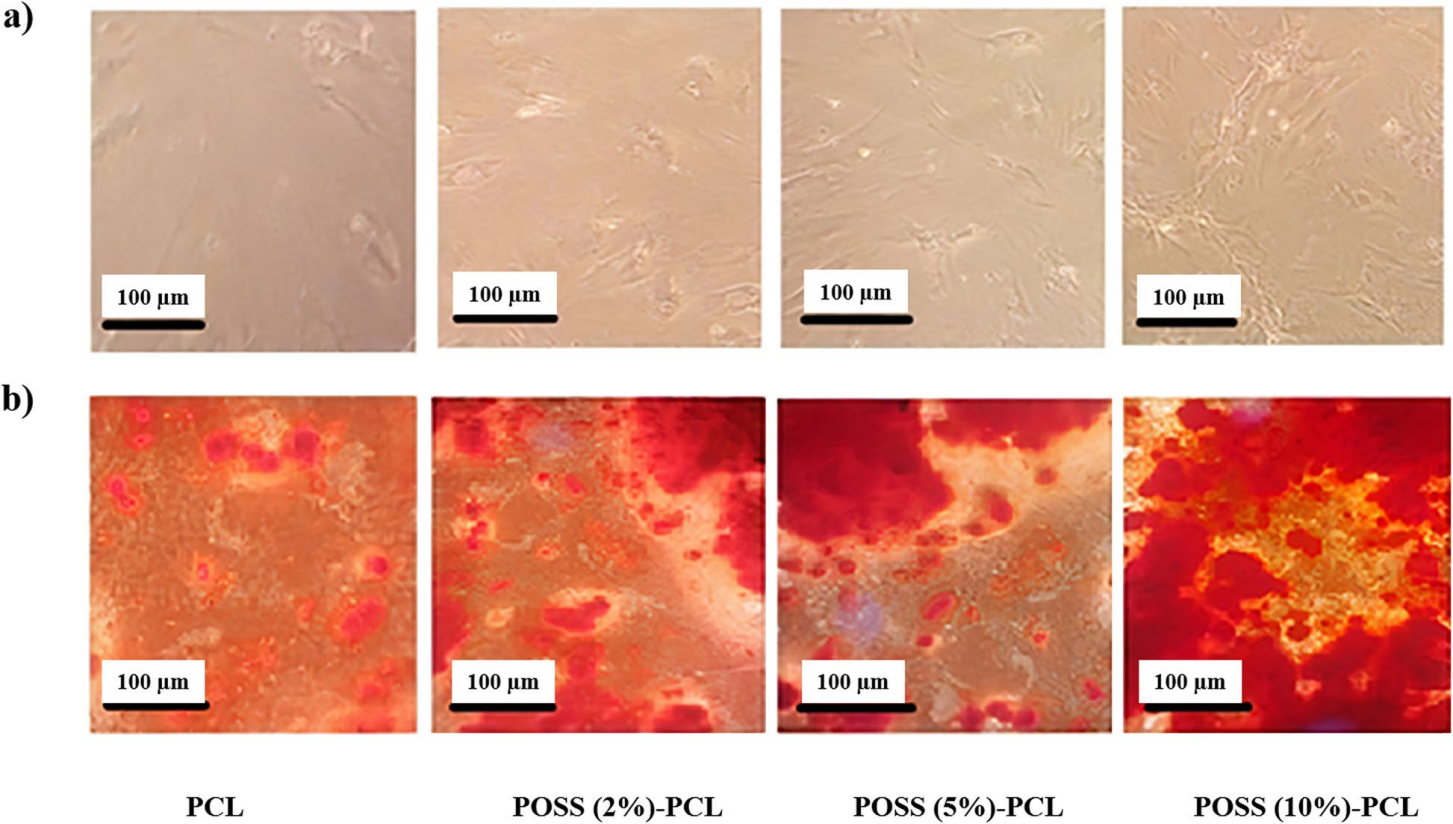
Alizarin red staining of MSCs under osteogenesis differentiation seeded on different concentrations of POSS in scaffolds of POSS-(PCL)_8_. More differentiation of MSCs was observed on POSS-PCL compared with PCL scaffolds

Alizarin red staining was used to measure the differentiation of MSCs and the induction/inhibitory effect of designed scaffolds of POSS-PCL. As Fig. 4 shows, the red-stained cells are osteo-cells because of calcium deposition.

### Detection of the population of apoptotic and non-apoptotic cells

**Fig. 5:**
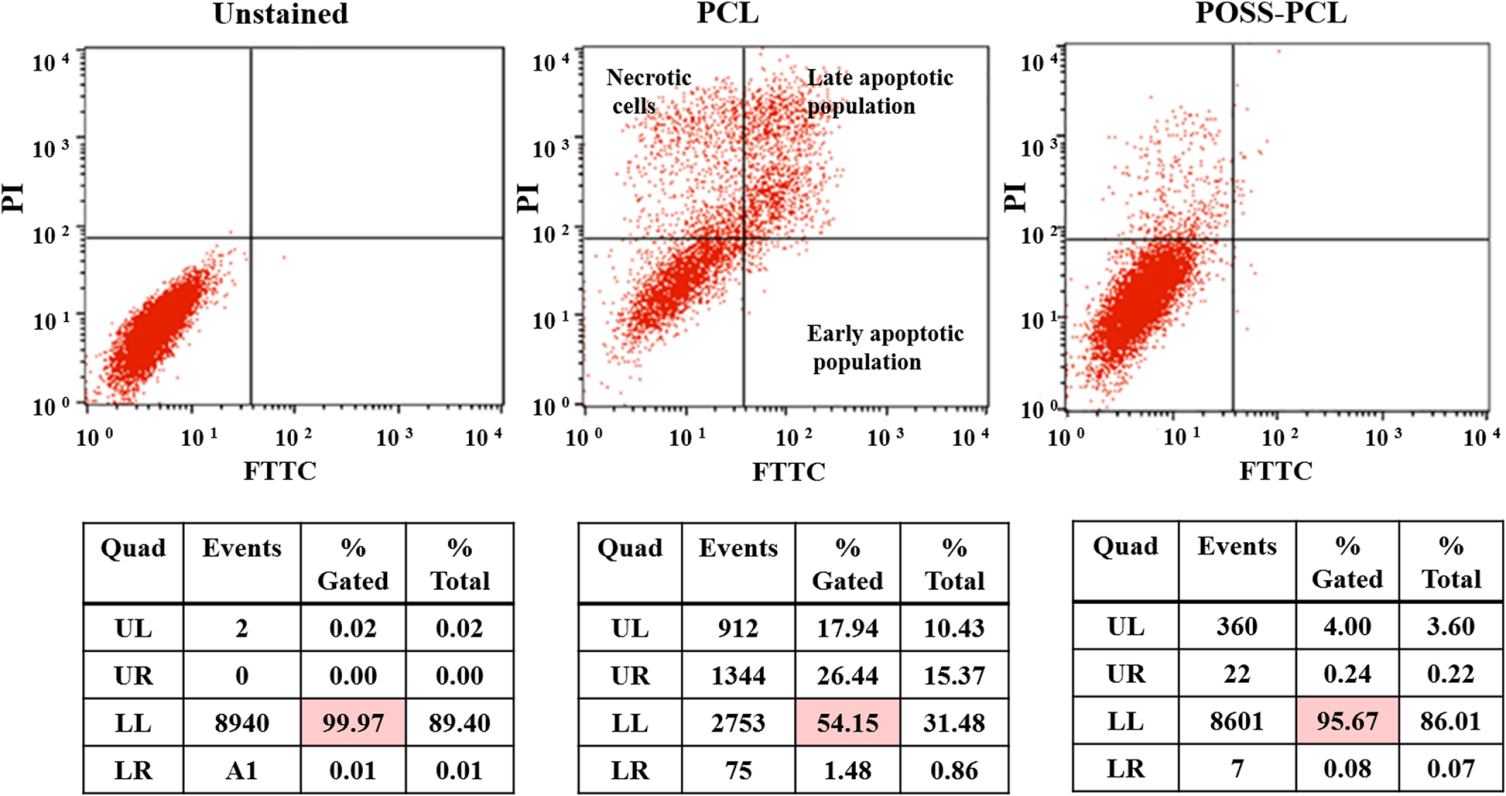
The cytotoxicity of PCL and POSS-PCL scaffolds on MSCs after 7 days of seeding. Annexin V- apoptotic detection assay by Flow cytometry and the results for three groups of control, the cells seeded on PCL scaffold and the cells seeded on POSS-PCL scaffolds. As the results show, about 50 percent of the cell’s protective effect was observed in POSS-PCL (95.67 %) compared with PCL (54.15 %) scaffolds

Annexin V-FITC and PI staining and flow cytometry analysis were used to detect the path of death of the studied cells for apoptosis and necrosis. The results of this study are shown in Fig 5a. Control group cells and untreated cells show survival of cells and cells of the treated group show increased apoptosis. The percentages of each group are shown in Fig 5b.

### Detection of intracellular stress imposing by nano-scaffolds

**Fig. 6:**
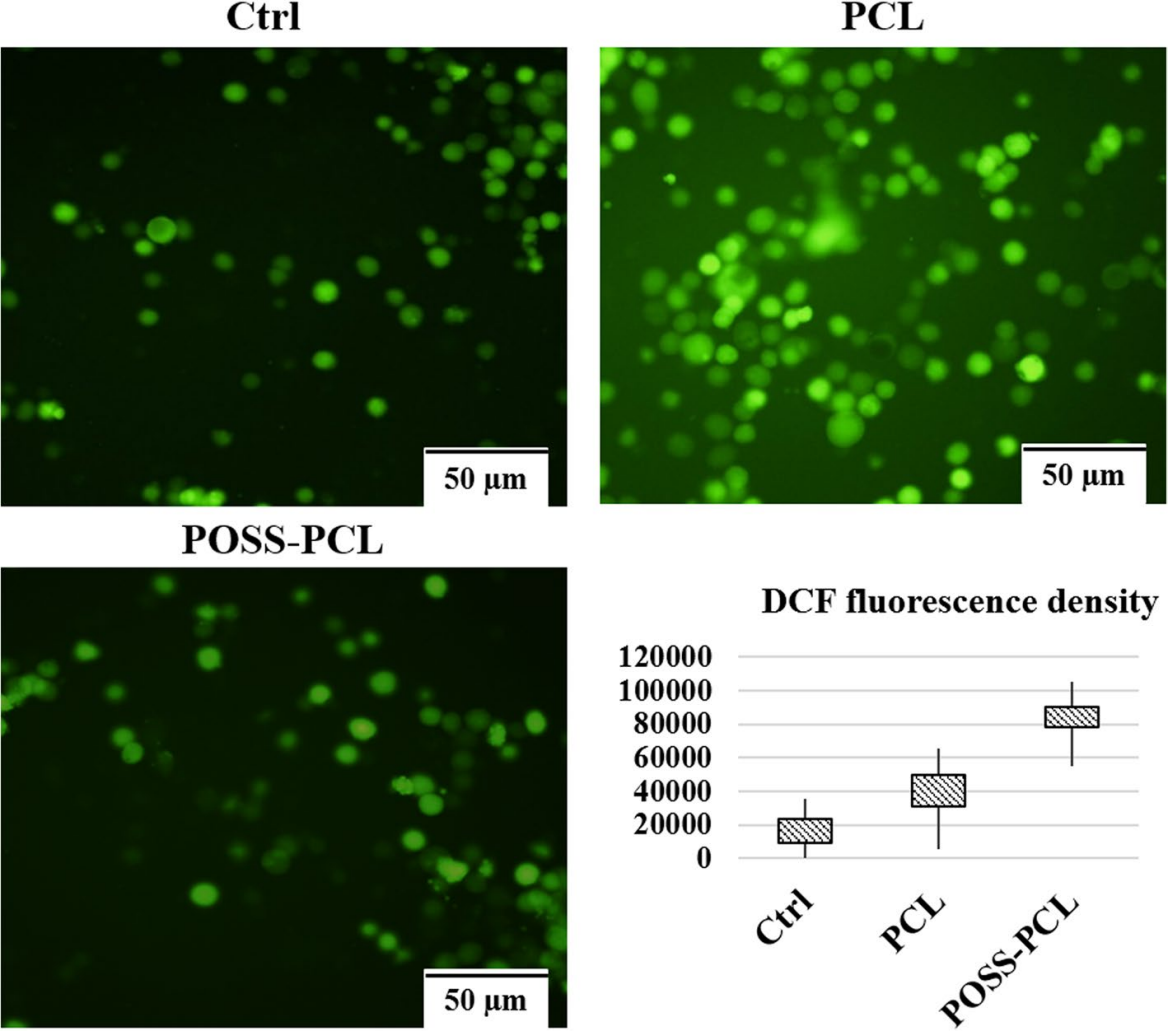
Intracellular ROS detection in MSCs seeded on scaffolds. The figures represent the fluorescence intensity of control MSCs, represent the cells cultured on PCL, and the cell seeded on POSS-PCL nanofiber. As the result shows, POSS-PCL compared with PCL nanofiber expos low fluorescent due to the low oxidative stresses

Fluorescence images of DCFH-stained MSCs seeded on POSS-PCL nanofibers were observed after 1 day. As shown, the rate of cell uptake and consequent green fluorescence intensity of POSS-PCL cells is higher than that of PCL cells, while there is no difference in fluorescence intensity between 2, 5, and 10% of POSS-PCL nanofibers (Fig. 6).

### RNA profile expression

**Fig. 7:**
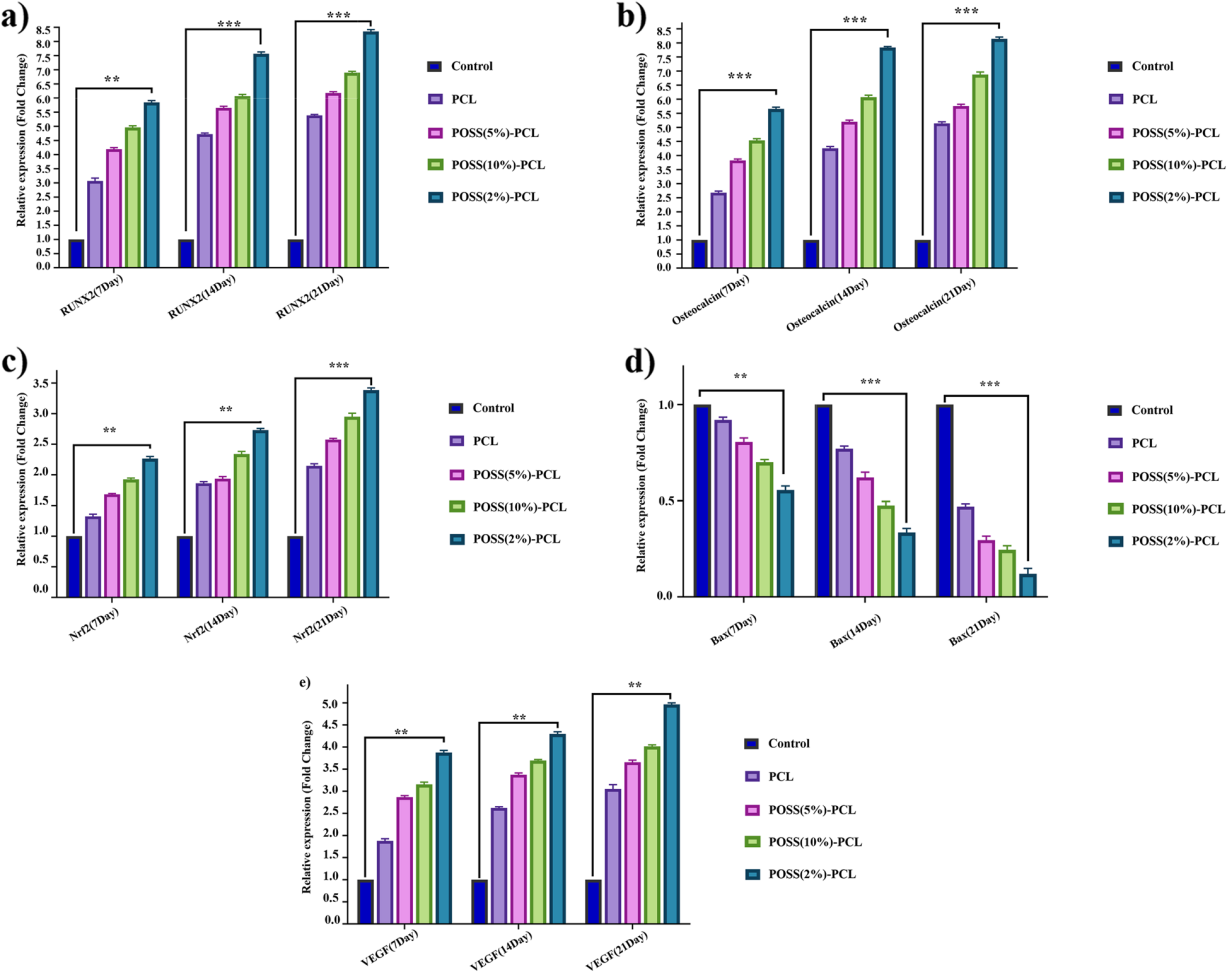
The mRNA profile of differentiation genes, apoptosis gene, antioxidant gene, and VEGF angiogenesis factor. the scaffolds must protect the cells from oxidative stress and apoptosis when injected into the harsh condition of damaged tissues and then, the cells must have a bio-functional role in secretome or differentiation. As the molecular studies show the upregulation of antioxidants gene, Nrf2, angiogenesis gene, BAX, and differentiation genes, including RUNX2 and Osteocalcin. No toxicity was observed in the POSS-PCL scaffold and BAX as the pro-apoptosis gene was downregulated. Data display the mean values ± SD of at minimum 3 autonomous experiments. **; Statistical significance (*P* <0.01) compared to the control (Ctrl)

Using specific primers, we performed Real- time testing for a set of genes associated with survival, stress, angiogenesis, and bone differentiation of cells exposed to POSS- PCL nano scaffolds with varying concentrations of POSS (Fig. 7).

### The effect of designed scaffold components in MSCs bio-function

#### The effect of scaffold component in MSCs differentiation

As Fig. 7 a show, the bone regeneration and differentiation of MSCs were not combatted by scaffolds. In addition, it was an inhaler of differentiation according to the upregulation of RUNX and Cal genes.

#### The effect of scaffold component in MSCs intracellular antioxidant pathway

Nuclear factor erythroid 2-related factor 2 (Nrf2) is a transcription activator that binds to the antioxidant response elements (ARE) in the promoter regions of target antioxidant genes and is critical for the coordinated up-regulation of genes in response to oxidative stress. As shown in Fig. 7, an increased level of Nrf2 was not seen in MSCs seeded on scaffolds.

#### Non-toxicity and biocompatibility of scaffolds

As Fig. 7 shows, any toxicity of scaffolds was not observed and the BAX gene as a pro-apoptotic gene was not upregulation in relation to control cells.

#### The therapeutic potentiality of MSCs seeded on scaffolds

The secretome of MSCs extremely influences cell therapy conclusions in injured tissues and as the calculations displayed, the designed scaffolds did not combat VEGF secretion but could upregulate VEGF (Fig. 7).

## Discussion

Nanofiber scaffolds can be formed by the electrospinning technique, and because of the nanometer fiber construction as well as the interconnection, great porosity, and monotony, they supply a good similarity to the physiological situation of the body [47, 48]. The electrospinning technique is the most extensively used scaffold manufacturing technique [49]. The cause for its great practice is the ease of practice for most ceramics, polymers, and metals, because of its low cost and the resemblance of the fabricated fibers with the body fibrous tissues [50]. In this study, POSS-PCL electrospun scaffolds were produced from two diverse polymers that include the polymers that have been investigated widely in bone tissue engineering. The same studies that have been studied so far are POSS-PCL and PCL-HA composites, and the advantage of this study over them is that POSS-PCL nanocomposite has gone one step further and been synthesized as nanofiber and was examined in bone regeneration [51]. Other similar works that can be mentioned in this regard are PCL/POSS, Gel-POSS and, Chitosan-hybrid POSS nanocomposites, which have been investigated and described the structure, biocompatibility, properties and morphology of these nanocomposites and the role of POSS in increasing the degradability of hydrogels [18, 52, 53]. The purpose of this study was comparison of osteoinductivity of POSS (2%)-PCL, POSS (5%)-PCL, POSS (10%)-PCL, and PCL nanofibers scaffolds manufactured by electrospinning by culturing of AD-MSCs on them. Morphological investigation of the manufactured nanofibers by SEM confirmed that POSS-PCL nanofibers scaffolds have approximately a construction with a random orientation and flat surface and porous. Outcomes confirmed that the viability of the cells on the POSS-PCL nanofibers scaffolds has not been significantly different. Stem cells were proliferated, growing, and expanding on the produced scaffolds that verified their fine biocompatibility. Then, to study the osteoinductivity of the manufactured nanofibers scaffolds, distinguished AD-MSCs were cultivated on the surface of POSS (2%)-PCL, POSS (5%)-PCL, POSS (10%)-PCL, and PCL scaffolds and then stem cells osteogenic differentiation potential was assessed at the protein and gene levels. Prior studies have described similar outcomes when using AD-MSCs for tissue engineering aims. It was perceived that AD-MSCs enhance the angiogenesis of the biodegradable implants. First, we assessed Alizarin red, DCFH, Annexin V-FITC, and PI staining of stem cells on POSS (2, 5, and 10%)-PCL and PCL scaffolds, which displayed cell differentiation and cell viability, and green fluorescence intensity on scaffolds of POSS-PCL is more than PCL. Bone-like POSS-PCL creation by deposition of Ca ions in body liquids is one of the beneficial features of polymeric biomaterials for practice in the structure of bone implants [54, 55]. Then, the expression level of four significant osteogenic gene markers such as Runx2, Nrf2, BAX, and VEGF was assessed in the distinguished AD-MSCs on POSS-PCL substrates. According to the outcomes gained from Alizarin red, DCFH, Annexin V-FITC, and PI assays, the expression level of osteogenic gene markers in AD-MSCs cultivated on POSS-PCL was meaningfully greater than in PCL groups, while expression diversities between AD-MSCs cultivated on POSS (2%)-PCL, POSS (5%)-PCL, and POSS (10%)-PCL scaffolds wasn’t significant. But with a rising percentage of POSS, the results become more favorable. The only study that is analogous in comparison to these POSS-PCL scaffolds is HA-PCL, which according to studies, in this comparison, POSS-PCL has more bone-producing features. This diversity between the osteoporosis of these two scaffolds can be correlated to the singular feature of POSS.

## Conclusions

POSS-PCL nanocomposites were synthesized by a hybrid combination of POSS and PCl polymer and the nanofibers were made from this nanocomposite using the electrospinning method. Mesenchymal Stem Cells (MSCs) were cultured on scaffolds. The results showed no observed cytotoxicity for these nanofibers and POSS-PCL nanofibers showed cell stress reduction, better cell viability, angiogenesis, and bone differentiation for POSS-PCL nanofibers when compared to PCL. Meanwhile, after trypsin and collecting the cells, the nanofibers were moved to the trash, because we did not see any problem in the stability of the nanofibers. And in all the plates, the nanofibers were not dissolved until the last step of collecting the cells and were completely preserved. This is also observed in the analysis of alizarin red. Images related to alizarin red analysis show the image of cell differentiation on stable nanofibers. The comparison days were 7, 14, and 21 days, respectively, which showed good cell adhesion and dispersion on scaffolds. In this study, the RUNX2 and Osteocalcin genes provide instructions for making a protein involved in the development and maintenance of the bones. The BAX gene expression profile was adjusted as a pro-apoptosis gene, meaning that the studied scaffold did not show any cytotoxicity. In the case of Nrf2, it can be said that by incremental adjustment, this antioxidant gene, and especially the super transcription factor, the scaffold, does not cause any oxidative stress in the cells. In the end, the primary therapeutic potentiality of MSCs, angiogenesis potentiality by VEGF factor, was evaluated after nesting in the scaffold. The result showed a significant increase in VEGF in cells nesting in our formulated matrix. The DCFH free radical detection system confirmed the Q-PCR result. Finally, the POSS-PCL nanofiber scaffold can be employed as an ideal scaffold in tissue engineering for bone regeneration.

## Data Availability

The data that support the findings of this study are available from the corresponding author, upon reasonable request.

## References

[1] Natarajan D, Ye Z, Wang L, Ge L, Pathak JL (2022). Rare earth smart nanomaterials for bone tissue engineering and implantology: Advances, challenges, and prospects. Bioeng Transl Med..

[2] Muzzio N, Moya S, Romero G (2021). Multifunctional scaffolds and synergistic strategies in tissue engineering and regenerative medicine. Pharmaceutics..

[3] Wei W, Dai H (2021). Articular cartilage and osteochondral tissue engineering techniques: Recent advances and challenges. Bioact Mater..

[4] Sahabi S, Jafari-Gharabaghlou D, Zarghami N (2022). A new insight into cell biological and biochemical changes through aging. Acta Histochem..

[5] Zamani R, Aval SF, Pilehvar-Soltanahmadi Y, Nejati-Koshki K, Zarghami N (2018). Recent advances in cell electrospining of natural and synthetic nanofibers for regenerative medicine. Drug Res..

[6] Augustine R, Dan P, Hasan A, Khalaf IM, Prasad P, Ghosal K (2021). Stem cell-based approaches in cardiac tissue engineering: Controlling the microenvironment for autologous cells. Biomed Pharmacother..

[7] Dadashpour M, Pilehvar-Soltanahmadi Y, Mohammadi SA, Zarghami N, Pourhassan-Moghaddam M, Alizadeh E (2018). Watercress-based electrospun nanofibrous scaffolds enhance proliferation and stemness preservation of human adipose-derived stem cells. Artif Cells Nanomed Biotechnol..

[8] Nejati K, Mehdi D, Ghareghomi S, Mostafavi E, Ebrahimi-Kalan A, Biglari A (2020). GDNF gene-engineered adipose-derived stem cells seeded Emu oil-loaded electrospun nanofibers for axonal regeneration following spinal cord injury. J Drug Deliv Sci Technol..

[9] Salehi AOM, Keshel SH, Sefat F, Tayebi L (2021). Use of polycaprolactone in corneal tissue engineering: A review. Mater Today Commun..

[10] Marew T, Birhanu G (2021). Three dimensional printed nanostructure biomaterials for bone tissue engineering. Regen Ther..

[11] Prigyai N, Chanmungkalakul S, Sukwattanasinitt M, Ervithayasuporn V (2021). Symmetry driven: the synthesis of co-substituent octasilsesquioxanes. New Journal of Chemistry.

[12] Kabra S, Tandon S, Kandasubramanian B. POSS nanocomposites for defense and space applications. InPolyhedral Oligomeric Silsesquioxane (POSS) Polymer Nanocomposites. Elsevier. 2021;81-498.

[13] Boccaleri E, Carniato F. Synthesis routes of poss. InPolymer/POSS Nanocomposites and Hybrid Materials. Cham: Springer; 2018. pp. 1-26.

[14] Januszewski R, Dutkiewicz M, Nowicki M, Kownacki I (2020). Synthesis and properties of hybrid materials obtained via additive cross-linking of liquid polybutadiene rubber with H-Si containing reagents. Polym Test.

[15] Loman-Cortes P, BinteHuq T, Vivero-Escoto JL (2021). Use of polyhedral oligomeric silsesquioxane (POSS) in drug delivery, photodynamic therapy and bioimaging. Molecules.

[16] Nowacka M, Makowski T, Kowalewska A (2020). Hybrid fluorescent poly (Silsesquioxanes) with amide-and triazole-containing side groups for light harvesting and cation sensing. Materials (Basel).

[17] Osaki M, Ito K, Ikemoto Y, Yamaguchi H, Chujo Y, Harada A (2020). Photoresponsive polymeric actuator cross-linked by an 8-armed polyhedral oligomeric silsesquioxane. Eur Polym J.

[18] Cobos M, Ramos JR, Guzmán DJ, Fernández MD, Fernández MJ (2018). PCL/POSS nanocomposites: Effect of POSS derivative and preparation method on morphology and properties. Polymers (Basel).

[19] Zhao B, Mei H, Zheng S (2020). Polyethylene telechelics with POSS termini: synthesis, morphologies and shape memory properties. Polymer Chemistry.

[20] Shi H, Yang J, Li Z, He C. Functionalized Polyhedral Oligomeric Silsesquioxanes (POSS) and Copolymers: Methods and Advances. Silicon Containing Hybrid Copolymers. 2020:63-96.

[21] Kong J, Tan BH, Lu X, Li Z, He C. Hybrid POSS Nanocomposites: An Overview of Material Toughening and Fire Retardancy. Silicon Containing Hybrid Copolymers. 2020:201-37.

[22] Ayandele E, Sarkar B, Alexandridis P (2012). Polyhedral oligomeric silsesquioxane (POSS)-containing polymer nanocomposites. Nanomaterials.

[23] Amna T, Hassan MS, El-Newehy MH, Alghamdi T, MoydeenAbdulhameed M, Khil M-S (2021). Biocompatibility Computation of Muscle Cells on Polyhedral Oligomeric Silsesquioxane-Grafted Polyurethane Nanomatrix. Nanomaterials.

[24] Nejati-Koshki K, Mortazavi Y, Pilehvar-Soltanahmadi Y, Sheoran S, Zarghami N (2017). An update on application of nanotechnology and stem cells in spinal cord injury regeneration. Biomed Pharmacother..

[25] Bharadwaz A, Jayasuriya AC (2020). Recent trends in the application of widely used natural and synthetic polymer nanocomposites in bone tissue regeneration. Mater Sci Eng C Mater Biol Appl.

[26] Jiang S, Wang M, He J (2021). A review of biomimetic scaffolds for bone regeneration: Toward a cell-free strategy. Bioeng Transl Med.

[27] Liu Z, Hu D, Huang L, Li W, Tian J, Lu L (2018). Simultaneous improvement in toughness, strength and biocompatibility of poly (lactic acid) with polyhedral oligomeric silsesquioxane. Chem Eng J.

[28] Jia L, Ma J, Gao D, Tait WR, Sun L (2019). A star-shaped POSS-containing polymer for cleaner leather processing. J Hazard Mater.

[29] Kausar A (2017). State-of-the-Art overview on polymer/POSS nanocomposite. Polym Plast Technol Eng.

[30] Kalia S, Pielichowski K, editors. Polymer/POSS Nanocomposites and Hybrid Materials: Preparation, Properties, Applications. Springer; 2018.

[31] Cobos M, Ramos JR, Guzmán DJ, Fernández MD, Fernández M (2019). PCL/POSS nanocomposites: Effect of POSS derivative and preparation method on morphology and properties. Polymers (Basel).

[32] Wang W, Lu Z, Li J, Bártolo P (2020). Engineering the biological performance of hierarchical nanostructured poly (ε-carpolactone) scaffolds for bone tissue engineering. CIRP Annals.

[33] Nezakati T, Tan A, Lim J, Cormia RD, Teoh SH (2019). Ultra-low percolation threshold POSS-PCL/graphene electrically conductive polymer: Neural tissue engineering nanocomposites for neurosurgery. Mater Sci Eng C Mater Biol Appl.

[34] Zhou H, Ye Q, Xu J (2017). Polyhedral oligomeric silsesquioxane-based hybrid materials and their applications. Mater Chem Front.

[35] Miltner HE, Watzeels N, Goffin A-L, Duquesne E, Benali S, Dubois P (2010). Quantifying the degree of nanofiller dispersion by advanced thermal analysis: application to polyester nanocomposites prepared by various elaboration methods. J Mater Chem..

[36] Hong JK, Cooke SL, Whittington AR, Roman M (2021). Bioactive cellulose nanocrystal-poly (ε-caprolactone) nanocomposites for bone tissue engineering applications. Front Bioeng Biotechnol.

[37] Teng S-Q, Jiang Z-G, Qiu Z-B (2020). Crystallization behavior and dynamic mechanical properties of Poly (ε-caprolactone)/Octaisobutyl-Polyhedral oligomeric silsesquioxanes composites prepared via different methods. Chin J Polym Sci.

[38] Cobos M, Ramos JR, Guzmán DJ, Fernández MD, Fernández MJ (2018). PCL/POSS nanocomposites: Effect of POSS derivative and preparation method on morphology and properties. Polymers..

[39] Hong JK, Cooke SL, Whittington AR, Roman M (2021). Bioactive cellulose nanocrystal-poly (ε-caprolactone) nanocomposites for bone tissue engineering applications. Front Bioeng Biotechnol..

[40] Teng S-Q, Jiang Z-G, Qiu Z-B (2020). Crystallization behavior and dynamic mechanical properties of poly (ε-caprolactone)/octaisobutyl-polyhedral oligomeric silsesquioxanes composites prepared via different methods. Chin J Polym Sci..

[41] Chen M, Zhang Y, Zhang W, Li J (2020). Polyhedral oligomeric silsesquioxane-incorporated gelatin hydrogel promotes angiogenesis during vascularized bone regeneration. ACS Appl Mater Interfaces.

[42] Li H, Shen S, Fu H, Wang Z, Li X, Sui X (2019). Immunomodulatory functions of mesenchymal stem cells in tissue engineering. Stem Cells Int.

[43] Ayala R, Zhang C, Yang D, Hwang Y, Aung A, Shroff SS (2011). Engineering the cell–material interface for controlling stem cell adhesion, migration, and differentiation. Biomaterials.

[44] Yorukoglu AC, Kiter A, Akkaya S, Satiroglu-Tufan NL, Tufan AC (2017). A concise review on the use of mesenchymal stem cells in cell sheet-based tissue engineering with special emphasis on bone tissue regeneration. Stem Cells Int.

[45] Kanaoka C (2019). Fine particle filtration technology using fiber as dust collection medium. KONA Powder Particle J.

[46] Pfaffl MW (2001). A new mathematical model for relative quantification in real-time RT–PCR. Nucleic Acids Res..

[47] Yao Y, Xu Y, Wang B, Yin W, Lu H (2018). Recent development in electrospun polymer fiber and their composites with shape memory property: a review. Pigment Resin Technol.

[48] Abazari MF, Hosseini Z, Karizi SZ, Norouzi S, Faskhoudi MA, Saburi E (2020). Different osteogenic differentiation potential of mesenchymal stem cells on three different polymeric substrates. Gene.

[49] Aydogdu A, Sumnu G, Sahin S (2019). Fabrication of gallic acid loaded Hydroxypropyl methylcellulose nanofibers by electrospinning technique as active packaging material. Carbohydr Polym.

[50] Rijal NP, Adhikari U, Khanal S, Pai D, Sankar J, Bhattarai N (2018). Magnesium oxide-poly (ε-caprolactone)-chitosan-based composite nanofiber for tissue engineering applications. Mater Sci Eng B.

[51] Liu F, Kang H, Liu Z, Jin S, Yan G, Sun Y (2021). 3D Printed multi-functional scaffolds based on poly (ε-caprolactone) and hydroxyapatite composites. Nanomaterials (Basel).

[52] Chen M, Zhang Y, Xie Q, Zhang W, Pan X, Gu P (2019). Long-term bone regeneration enabled by a polyhedral oligomeric silsesquioxane (POSS)-enhanced biodegradable hydrogel. ACS Biomater Sci Eng.

[53] Tamburaci S, Tihminlioglu F (2020). Chitosan-hybrid poss nanocomposites for bone regeneration: The effect of poss nanocage on surface, morphology, structure and in vitro bioactivity. Int J Biol Macromol.

[54] Satpathy A, Pal A, Sengupta S, Das A, Hasan M, Ratha I (2019). Bioactive nano-hydroxyapatite doped electrospun PVA-chitosan composite nanofibers for bone tissue engineering applications. J Indian Inst Sci.

[55] Naderi N, Griffin M, Mosahebi A, Butler P, Seifalian A (2020). Adipose derived stem cells and platelet rich plasma improve the tissue integration and angiogenesis of biodegradable scaffolds for soft tissue regeneration. Mol Biol Rep..

